# (*E*)-1,2-Bis(3-bromo-4-methyl­phen­yl)ethene

**DOI:** 10.1107/S1600536807067645

**Published:** 2008-01-04

**Authors:** René T. Boeré, Steven J. Robbins

**Affiliations:** a Department of Chemistry and Biochemistry, University of Lethbridge, Lethbridge, AB, Canada T1K 3M4

## Abstract

In the structure of the title compound, C_16_H_14_Br_2_, the central C=C bond length is 1.329 (4) Å and the two benzene rings are approximately coplanar with the double bond, with twist angles of 7.5 (2) and 13.6 (2)°.

## Related literature

For related literature, see: Daik *et al.* (1998 ▶); Harada & Ogawa *et al.* (2004 ▶); Ogawa *et al.* (1992 ▶); Mallory *et al.* (2001 ▶).
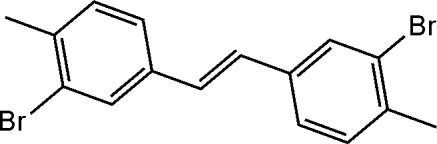


## Experimental

### 

#### Crystal data

C_16_H_14_Br_2_
*M* *_r_* = 366.09
Monoclinic, 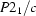

*a* = 6.3301 (4) Å
*b* = 7.6499 (5) Å
*c* = 28.164 (2) Å
β = 91.208 (1)°
*V* = 1363.55 (16) Å^3^
*Z* = 4
Mo *K*α radiation
μ = 5.92 mm^−1^
*T* = 173 (2) K
0.27 × 0.19 × 0.10 mm

#### Data collection

Bruker APEXII CCD area-detector diffractometer
Absorption correction: multi-scan (*SADABS*; Sheldrick, 2004 ▶) *T*_min_ = 0.303, *T*_max_ = 0.578
14025 measured reflections
2793 independent reflections
2393 reflections with *I* > 2σ(*I*)
*R*_int_ = 0.027

#### Refinement

*R*[*F* ^2^ > 2σ(*F* ^2^)] = 0.025
*wR*(*F* ^2^) = 0.054
*S* = 1.08
2793 reflections
165 parameters
H-atom parameters constrained
Δρ_max_ = 0.42 e Å^−3^
Δρ_min_ = −0.32 e Å^−3^

### 

Data collection: *APEX2* (Bruker, 2006 ▶); cell refinement: *APEX2*; data reduction: *SAINT-Plus* (Bruker, 2006 ▶); program(s) used to solve structure: *SHELXS97* (Sheldrick, 1990 ▶); program(s) used to refine structure: *SHELXTL* (Sheldrick, 2003 ▶); molecular graphics: *Mercury* (Macrae *et al.*, 2006 ▶); software used to prepare material for publication: *publCIF* (Westrip, 2008 ▶).

## Supplementary Material

Crystal structure: contains datablocks I, global. DOI: 10.1107/S1600536807067645/pv2061sup1.cif

Structure factors: contains datablocks I. DOI: 10.1107/S1600536807067645/pv2061Isup2.hkl

Additional supplementary materials:  crystallographic information; 3D view; checkCIF report

## Figures and Tables

**Fig. 1.**
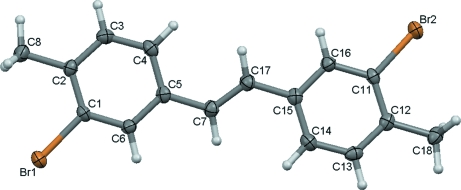
A view of (I) plotted with displacement ellipsoids at 50% probability level.

**Fig. 2.**
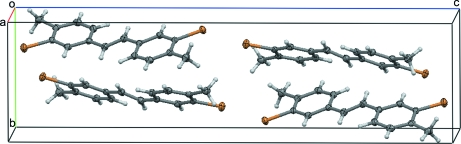
Unit-cell contents of (I) showing the herringbone arrangement of slipped-stacks with T-contacts between registers.

## supplementary crystallographic information

### Comment

The title compound, (I) (Fig. 1), was prepared by a Ti catalyzed McMurray coupling (Mallory *et al.*, 2001) with 99% E selectivity. The almost-planar molecules pack (Fig. 2) in slipped stacks with T-contacts typical for aromatic molecules. The only other isomeric stilbene for which a structure has been reported is *Z*-1,2-bis-(4-bromophenyl)-1,2-dimethylethene (Daik *et al.*, 1998) for which C=C is 1.330 (10) and 1.344 (10)Å (for two independent molecules in an asymmetric unit). Unlike in I, the phenyl rings in this compound are twisted almost orthogonal to the double bond, perhaps because of steric interactions between methyl and phenyl groups. More structurally comparable alkenes include *E*-1,2-bis-(2,4-dimethylphenyl)ethene and *E*-1,2-bis-(2,4,5-trimethylphenyl)ethene (Ogawa *et al.*, 1992) for which C=C are 1.320 (4) and 1.327 (3) Å, respectively. A detailed study of geometric distortions in *trans*-stilbene has recently been published (Harada & Ogawa, 2004).

### Experimental

At 273 K under N_2_, 0.18 ml (1.6 mmol) of TiCl_4_ was stirred with 0.18 g (2.8 mmol) Zn dust in 25 ml of dry THF. To this mixture was added 0.25 g (1.3 mmol) of 3-bromo-4-methylbenzaldehyde and refluxed for 4 h before being quenched with 25 ml of 1.0 *M* HCl. After extracting with hexanes the organic phase was washed with brine solution and dried over MgSO_4_. Removal of solvent resulted in a white powder that was recrystallized from ethyl acetate to give 0.11 g (yield = 58%) of the desired product as colorless crystals.

### Refinement

The H-atoms were included in the refinements at geometrically idealized positions with C—H distances 0.95 and 0.98 Å for non-methyl and methyl type H-atoms, respectively; *U*_iso_ values were 1.2*U*_eq_ of the carrier atom or 1.5*U*_eq_ for the non-methyl and methyl groups, respectively.

### Crystal data

**Table:**

C_16_H_14_Br_2_	*Z* = 4
*M**_r_* = 366.09	*F*_000_ = 720
Monoclinic, *P*2_1_/*c*	*D*_x_ = 1.783 Mg m^−^^3^
Hall symbol: -P2ybc	Melting point: 424.75 K
*a* = 6.3301 (4) Å	Mo *K*α radiation λ = 0.71073 Å
*b* = 7.6499 (5) Å	µ = 5.92 mm^−^^1^
*c* = 28.164 (2) Å	*T* = 173 (2) K
β = 91.208 (1)º	Prism, colourless
*V* = 1363.55 (16) Å^3^	0.27 × 0.19 × 0.10 mm

### Data collection

**Table:**

Bruker APEXII CCD area-detector diffractometer	2793 independent reflections
Monochromator: graphite	2393 reflections with *I* > 2σ(*I*)
*T* = 173(2) K	*R*_int_ = 0.027
*P* = 101 kPa	θ_max_ = 26.4º
φ and ω scans	θ_min_ = 2.8º
Absorption correction: multi-scan(SADABS; Sheldrick, 2004)	*h* = −7→7
*T*_min_ = 0.303, *T*_max_ = 0.578	*k* = −9→9
14025 measured reflections	*l* = −35→35

### Refinement

**Table:**

Refinement on *F*^2^	Secondary atom site location: difference Fourier map
Least-squares matrix: full	Hydrogen site location: inferred from neighbouring sites
*R*[*F*^2^ > 2σ(*F*^2^)] = 0.025	H-atom parameters constrained
*wR*(*F*^2^) = 0.054	*w* = 1/[σ^2^(*F*_o_^2^) + (0.0179*P*)^2^ + 1.6865*P*] where *P* = (*F*_o_^2^ + 2*F*_c_^2^)/3
*S* = 1.09	(Δ/σ)_max_ = 0.001
2793 reflections	Δρ_max_ = 0.43 e Å^−^^3^
165 parameters	Δρ_min_ = −0.32 e Å^−^^3^
Primary atom site location: structure-invariant direct methods	Extinction correction: none

### Special details

**Table:**

Geometry. All e.s.d.'s (except the e.s.d. in the dihedral angle between two l.s. planes) are estimated using the full covariance matrix. The cell e.s.d.'s are taken into account individually in the estimation of e.s.d.'s in distances, angles and torsion angles; correlations between e.s.d.'s in cell parameters are only used when they are defined by crystal symmetry. An approximate (isotropic) treatment of cell e.s.d.'s is used for estimating e.s.d.'s involving l.s. planes.
Refinement. Refinement of *F*^2^ against ALL reflections. The weighted *R*-factor *wR* and goodness of fit *S* are based on *F*^2^, conventional *R*-factors *R* are based on *F*, with *F* set to zero for negative *F*^2^. The threshold expression of *F*^2^ > σ(*F*^2^) is used only for calculating *R*-factors(gt) *etc*. and is not relevant to the choice of reflections for refinement. *R*-factors based on *F*^2^ are statistically about twice as large as those based on *F*, and *R*- factors based on ALL data will be even larger.

### Fractional atomic coordinates and isotropic or equivalent isotropic displacement parameters (Å^2^)

**Table:**

	*x*	*y*	*z*	*U*_iso_*/*U*_eq_	
Br1	0.08255 (4)	0.93057 (4)	0.562520 (9)	0.02689 (8)	
C1	0.0145 (4)	0.8529 (3)	0.62456 (8)	0.0197 (5)	
C2	−0.1806 (4)	0.7739 (3)	0.63183 (9)	0.0217 (5)	
C3	−0.2158 (4)	0.7151 (3)	0.67779 (10)	0.0241 (6)	
H3	−0.3476	0.6620	0.6844	0.029*	
C4	−0.0673 (4)	0.7309 (3)	0.71400 (9)	0.0237 (6)	
H4	−0.0972	0.6867	0.7446	0.028*	
C5	0.1280 (4)	0.8119 (3)	0.70600 (9)	0.0213 (6)	
C6	0.1650 (4)	0.8749 (3)	0.66049 (9)	0.0212 (5)	
H6	0.2939	0.9332	0.6541	0.025*	
C7	0.2912 (4)	0.8315 (4)	0.74343 (9)	0.0241 (6)	
H7	0.4101	0.9015	0.7361	0.029*	
C8	−0.3452 (4)	0.7506 (4)	0.59307 (10)	0.0282 (6)	
H8A	−0.2872	0.6792	0.5676	0.034*	
H8B	−0.3865	0.8653	0.5804	0.034*	
H8C	−0.4693	0.6922	0.6060	0.034*	
Br2	0.44176 (4)	0.73105 (4)	0.971236 (9)	0.02871 (8)	
C11	0.5297 (4)	0.7870 (3)	0.90906 (9)	0.0205 (5)	
C12	0.7254 (4)	0.8675 (3)	0.90274 (9)	0.0218 (5)	
C13	0.7768 (4)	0.9044 (3)	0.85612 (10)	0.0243 (6)	
H13	0.9102	0.9558	0.8501	0.029*	
C14	0.6420 (4)	0.8695 (4)	0.81816 (9)	0.0253 (6)	
H14	0.6834	0.8991	0.7869	0.030*	
C15	0.4454 (4)	0.7913 (3)	0.82503 (9)	0.0218 (6)	
C16	0.3931 (4)	0.7476 (3)	0.87155 (9)	0.0224 (6)	
H16	0.2629	0.6906	0.8774	0.027*	
C17	0.2894 (4)	0.7613 (4)	0.78658 (9)	0.0250 (6)	
H17	0.1761	0.6842	0.7932	0.030*	
C18	0.8708 (4)	0.9153 (4)	0.94351 (10)	0.0279 (6)	
H18A	0.8014	1.0011	0.9638	0.033*	
H18B	1.0011	0.9656	0.9313	0.033*	
H18C	0.9047	0.8104	0.9621	0.033*	

### Atomic displacement parameters (Å^2^)

**Table:**

	*U*^11^	*U*^22^	*U*^33^	*U*^12^	*U*^13^	*U*^23^
Br1	0.02994 (15)	0.03138 (16)	0.01930 (14)	−0.00196 (12)	−0.00088 (10)	0.00574 (11)
C1	0.0243 (14)	0.0192 (13)	0.0157 (12)	0.0034 (11)	0.0018 (10)	0.0006 (10)
C2	0.0207 (13)	0.0217 (14)	0.0226 (13)	0.0025 (11)	−0.0021 (10)	−0.0016 (11)
C3	0.0214 (13)	0.0219 (14)	0.0291 (15)	0.0000 (11)	0.0031 (11)	0.0012 (11)
C4	0.0295 (15)	0.0221 (14)	0.0196 (13)	0.0031 (11)	0.0033 (11)	−0.0001 (11)
C5	0.0252 (14)	0.0196 (13)	0.0190 (13)	0.0025 (11)	0.0001 (11)	−0.0042 (10)
C6	0.0193 (13)	0.0224 (14)	0.0218 (13)	−0.0019 (11)	0.0014 (10)	−0.0017 (11)
C7	0.0253 (14)	0.0256 (15)	0.0213 (14)	0.0010 (11)	0.0001 (11)	−0.0028 (11)
C8	0.0247 (14)	0.0314 (16)	0.0283 (15)	−0.0022 (12)	−0.0052 (11)	−0.0001 (12)
Br2	0.03120 (16)	0.03709 (17)	0.01781 (14)	−0.00261 (12)	−0.00030 (11)	0.00279 (12)
C11	0.0259 (14)	0.0184 (13)	0.0172 (13)	0.0044 (11)	0.0003 (10)	0.0014 (10)
C12	0.0221 (13)	0.0169 (13)	0.0262 (14)	0.0042 (10)	−0.0029 (11)	−0.0014 (11)
C13	0.0192 (13)	0.0218 (14)	0.0321 (15)	0.0008 (11)	0.0027 (11)	0.0016 (11)
C14	0.0298 (15)	0.0249 (14)	0.0213 (14)	0.0026 (12)	0.0030 (11)	0.0018 (11)
C15	0.0251 (14)	0.0185 (13)	0.0217 (13)	0.0028 (11)	−0.0017 (11)	−0.0011 (10)
C16	0.0216 (13)	0.0216 (14)	0.0240 (14)	−0.0008 (11)	−0.0008 (10)	0.0009 (11)
C17	0.0287 (15)	0.0243 (14)	0.0218 (14)	−0.0022 (12)	−0.0013 (11)	−0.0023 (11)
C18	0.0272 (15)	0.0248 (15)	0.0314 (15)	−0.0020 (12)	−0.0052 (12)	−0.0019 (12)

### Geometric parameters (Å, °)

**Table:**

Br1—C1	1.904 (2)	Br2—C11	1.898 (3)
C1—C6	1.386 (3)	C11—C16	1.385 (4)
C1—C2	1.393 (4)	C11—C12	1.398 (4)
C2—C3	1.393 (4)	C12—C13	1.389 (4)
C2—C8	1.504 (4)	C12—C18	1.502 (4)
C3—C4	1.378 (4)	C13—C14	1.380 (4)
C3—H3	0.9500	C13—H13	0.9500
C4—C5	1.405 (4)	C14—C15	1.398 (4)
C4—H4	0.9500	C14—H14	0.9500
C5—C6	1.394 (4)	C15—C16	1.399 (4)
C5—C7	1.468 (4)	C15—C17	1.468 (4)
C6—H6	0.9500	C16—H16	0.9500
C7—C17	1.329 (4)	C17—H17	0.9500
C7—H7	0.9500	C18—H18A	0.9800
C8—H8A	0.9800	C18—H18B	0.9800
C8—H8B	0.9800	C18—H18C	0.9800
C8—H8C	0.9800		
			
C6—C1—C2	122.9 (2)	C16—C11—C12	122.7 (2)
C6—C1—Br1	117.82 (19)	C16—C11—Br2	117.7 (2)
C2—C1—Br1	119.25 (19)	C12—C11—Br2	119.67 (19)
C3—C2—C1	115.9 (2)	C13—C12—C11	115.9 (2)
C3—C2—C8	120.9 (2)	C13—C12—C18	121.4 (2)
C1—C2—C8	123.2 (2)	C11—C12—C18	122.7 (2)
C4—C3—C2	122.6 (3)	C14—C13—C12	122.7 (3)
C4—C3—H3	118.7	C14—C13—H13	118.7
C2—C3—H3	118.7	C12—C13—H13	118.7
C3—C4—C5	120.6 (2)	C13—C14—C15	120.9 (2)
C3—C4—H4	119.7	C13—C14—H14	119.6
C5—C4—H4	119.7	C15—C14—H14	119.6
C6—C5—C4	117.7 (2)	C14—C15—C16	117.5 (2)
C6—C5—C7	119.7 (2)	C14—C15—C17	123.5 (2)
C4—C5—C7	122.6 (2)	C16—C15—C17	119.0 (2)
C1—C6—C5	120.2 (2)	C11—C16—C15	120.4 (2)
C1—C6—H6	119.9	C11—C16—H16	119.8
C5—C6—H6	119.9	C15—C16—H16	119.8
C17—C7—C5	126.6 (3)	C7—C17—C15	126.4 (3)
C17—C7—H7	116.7	C7—C17—H17	116.8
C5—C7—H7	116.7	C15—C17—H17	116.8
C2—C8—H8A	109.5	C12—C18—H18A	109.5
C2—C8—H8B	109.5	C12—C18—H18B	109.5
H8A—C8—H8B	109.5	H18A—C18—H18B	109.5
C2—C8—H8C	109.5	C12—C18—H18C	109.5
H8A—C8—H8C	109.5	H18A—C18—H18C	109.5
H8B—C8—H8C	109.5	H18B—C18—H18C	109.5
			
Br2—C11—C12—C18	−1.0 (3)	C6—C5—C7—C17	−171.7 (3)
Br1—C1—C2—C8	−1.7 (4)	C16—C15—C17—C7	162.7 (3)
C4—C5—C7—C17	8.2 (4)	C5—C7—C17—C15	−175.4 (2)
C14—C15—C17—C7	−14.1 (4)		
